# Endovascular treatment in patients with carotid artery dissection and intracranial occlusion: a systematic review

**DOI:** 10.1007/s00234-017-1850-y

**Published:** 2017-06-03

**Authors:** Jan W. Hoving, Henk A. Marquering, Charles B. L. M. Majoie

**Affiliations:** 1 Department of Radiology, Academic Medical Center, University of Amsterdam, Amsterdam, The Netherlands; 2 Department of Biomedical Engineering and Physics, Academic Medical Center, University of Amsterdam, Amsterdam, The Netherlands

**Keywords:** Stroke, Internal carotid artery, Dissection, Thrombectomy, Stent

## Abstract

**Purpose:**

Recently, multiple randomised controlled trials showed efficacy of endovascular treatment over traditional care in patients with acute ischemic stroke due to an intracranial anterior circulation occlusion. Internal carotid artery (ICA) dissection with a concomitant intracranial occlusion is a rare but important cause of severe acute ischemic stroke. Although this subtype of acute ischemic stroke is mostly treated with endovascular treatment, treatment outcomes are still sparsely studied. This study assesses the clinical outcome and reperfusion rates by means of a systematic review.

**Methods:**

Electronic databases of PubMed, EMBASE and Web of Science were searched up to October 1, 2016 for articles describing endovascular treatment in patients with intracranial artery occlusion and ICA dissection.

**Results:**

Sixteen studies were included in the analysis. Most studies showed favourable outcome and successful reperfusion. However, most included studies had a high risk of bias.

**Conclusion:**

In the reviewed studies, endovascular treatment in patients with ICA dissection and concomitant proximal intracranial occlusion was associated with favourable outcome. This could point in the direction of endovascular treatment being a beneficial treatment method for these patients. However, this review has only taken data of a limited group of patients into account. A pooled analysis of patients from recently published endovascular treatment trials and running registries is therefore recommended.

**Electronic supplementary material:**

The online version of this article (doi:10.1007/s00234-017-1850-y) contains supplementary material, which is available to authorized users.

## Introduction

Internal carotid artery (ICA) dissection is a major cause of acute ischemic stroke (AIS) in the young, responsible for 10–25% of all strokes occurring in this population [1], whereas this severe cause of AIS only account for up to 2% in the total stroke population [2]. Before the introduction of intraarterial therapy, only few patients with extracranial ICA dissection together with an intracranial vessel occlusion were reported to fully recover after intravenous administration of rt-PA [3]. Over the past years, multiple studies on alternative ways of treatment such as intravenous fibrinolysis combined with endovascular treatment have been performed [4, 5]. As a result of the publication of multiple randomised controlled trials (MR CLEAN, ESCAPE, REVASCAT, SWIFT PRIME, EXTEND-IA, THRACE and PISTE) [6–12], intraarterial treatment has been proven effective over traditional treatment and has become the new standard for exclusive proximal intracranial occlusions. However, although patients with ICA dissection in combination with concomitant intracranial vessel occlusion are mostly treated with endovascular treatment, the clinical outcome and reperfusion rates are still sparsely studied, and therefore, this type of treatment is still unproven to be beneficial.

In this review, we assessed outcome for patients with ICA dissection in combination with intracranial vessel occlusion after use of endovascular treatment. We searched the available literature for cases describing patients with ICA dissection in combination with intracranial occlusion who received endovascular treatment and analysed clinical outcome and reperfusion rates.

## Methods

### Literature search

The online databases of PubMed, EMBASE and Web of Science were searched for data up to October 1, 2016. We used the following key words and MeSH terms: ‘stroke’, ‘thrombectomy’, ‘carotid dissection’, ‘intraarterial’, ‘endovascular treatment’ and ‘endovascular therapy’. After completion of the search, the filter ‘Humans’ was used for all of the articles found in the three databases. Articles not written in English were excluded from the search.

### Study selection

To meet the inclusion criteria for this systematic review, studies should have described patients with both ICA dissection and proximal intracranial occlusion, either as total sample of patients or as a subgroup, were included.

Subsequently, full-text screening of the articles was performed. Studies that only described patients with only internal carotid artery occlusion, exclusive ICA dissection, vertebrobasilar occlusion or dissection and patients who had exclusively received intravenous thrombolysis were excluded. Studies without specific dissection data, useful outcome parameters and description of the performed endovascular procedure were also not included in this review. Finally, conference abstracts were excluded.

### Data extraction

Data on study design, year of publication, country of origin, number of included patients and type of procedure were extracted. Patient characteristics such as gender, age and locations of both extracranial dissection together with the intracranial occlusion were recorded. We also extracted the following data: time to treatment (TTT), outcome rates graded as 90-day mRS, outcome rates graded as 90-day NIHSS and reperfusion and graded with the TICI score [13]. Reperfusion data was divided into the following subgroups: TICI 0–1, TICI 2a and TICI 2b-3.

To clearly denote the procedures for ICA dissection and intracranial occlusion that have been performed, the treatment options are represented in several subclasses. Procedures of ICA dissection treatment were divided into two subclasses: (1) permanent stent placement in ICA dissection and (2) no stenting of the ICA dissection. Procedures of intracranial occlusion treatment were divided into four subclasses: (1) use of intraarterial (IA) thrombolytics, (2) use of a Merci retriever, (3) use of a Penumbra device and (4) use of a Solitaire stent retriever. Finally, any complications or unexpected additional interventions during treatment were noted.

### Assessment of quality

The risk of bias was determined using an adapted version of the Cochrane Collaboration’s Risk of Bias Tool [14]**.** This tool represents a domain-based evaluation, which critically assesses various domains. These domains are sequence generation, blinding of participants and personnel, blinding of outcome assessment, incomplete outcome data, selective outcome reporting and ‘other issues’. The risk of bias for each domain was classified as ‘high’, ‘low’ or ‘unclear’.

### Study outcomes

In the studies included in this review, reperfusion was graded with the TICI score [13]. Accordingly, we defined TICI 2b-3 as successful reperfusion. In cases where reperfusion was described as favourable, we defined this as TICI 2b to prevent overestimation of reperfusion rates. Functional outcome was graded with 90-day mRS and 90-day NIHSS. When studies described both parameters, we chose to present both 90-day NIHSS and 90-day mRS to preserve all found outcome data. Favourable outcome was defined as a mRS score ≤2 and a NIHSS score ≤1 or improvement by at least 11 points from baseline [15].

## Results

### Search results

The initial search resulted in 351 studies. After removing 29 duplicates and 32 articles that were not accessible online, 290 articles remained. After screening on title and abstract, 254 articles were excluded based on relevancy, leaving 36 articles for full-text screening. Finally, 16 articles were included in this literature review. Most frequent reasons for exclusion were no clear description of the use of endovascular treatment and absence of clear outcome data.

### Study characteristics

Results of the risk of bias analysis are presented online as a supplementary table and as supplementary figures (see Online Resources 1, 2 and 3). All included studies were retrospective case reports or case series and therefore associated with an overall high risk of bias. There were only three domains in which reviewed studies scored a low risk of bias. These domains were ‘blinding outcome assessment’, ‘incomplete outcome data’ and ‘selective reporting’. Studies were only scored a low risk of bias if they were not likely to be influenced by lack of blinding, did not miss any outcome data and did not selectively reported their results.

### Procedural data

In most cases, procedures of ICA dissection treatment and intracranial occlusion treatment were combined to treat both abnormalities effectively. Seven studies described a cervical origin of the ICA dissection, whereas there was one study that described the case of an intracranial ICA dissection. We found that 12 studies exclusively described procedures in which the ICA dissection was addressed first. Furthermore, two studies described a treatment strategy with first addressing the intracranial occlusion before stenting the ICA dissection. In two studies, it was not clearly described which occlusive pathology was addressed first.

### Procedures of ICA dissection treatment

Permanent stent placement in ICA dissection together with antiplatelet therapy was described in ten studies [1, 16–24]. Permanent stents used in these cases were Carotid Wallstent [1, 16, 19, 20, 22, 24], Enterprise stent [17, 21], Wingspan stent [21], Acculink stent [25] and Leo stent [18, 24]. In four studies [2, 26–28], no stents were used to treat the ICA dissections.

### Procedures of intracranial occlusion treatment

Eleven studies showed IA thrombolytics as a supplemental treatment for the intracranial occlusion next to permanent stenting of the ICA dissection [1, 2, 16, 18–21, 23, 25, 27, 28]. In the two studies which did not describe the use of stents to treat the ICA dissection [27, 28], one case described treatment of the intracranial occlusion with IA rt-PA [28] and one case described use of IA urokinase as treatment for the intracranial occlusion [27].

One study [2] described the use of a Merci stent retriever to treat the intracranial occlusion. Two studies [25, 26] described the use of a Penumbra thromboaspiration system without a direct aspiration first-pass technique (ADAPT). Treatment of the intracranial occlusion with a Solitaire stent retriever was described in three studies [22, 27, 29]. In most cases, a combination of the approaches which are mentioned above is performed. For a more comprehensive description of performed procedures, see Table 1.

**Table 1:** Patient and study characteristics

Study	Study design	Vessel dissected	Additional thrombus	TTT (h)	Procedure of ICA dissection treatment	Procedure of intracranial occlusion treatment	Recanalisation (TICI)	90-day mRS	0-day NIHSS	90-day NIHSS	Favourable clinical outcome (FCO)
Baumgartner et al. [16]	Case-control	L ICA (4 points)	MCA	4.2	Carotid Wallstent	IA urokinase	N.A.	N.A.	18	8	+ 4/4
Bulsara et al. [28]	Case report	L ICA	L M2	1.5	None	IA rt-PA (2 mg) + 4 mg systemic rt-PA	N.A.	N.A.	20	0	+
Sainz de la Maza et al. [27] (2 cases)	Case report	L ICA	MCA	4.0	None	IA urokinase	3	0	N.A.	N.A.	+
	Case report	R ICA	R MCA	8.0	None	Solitaire stent retriever	3	N.A.	18	1	+
Fields et al. [2] (8 cases)	Case series	L ICA	L M1	9.4	Stent	Merci stent retriever + heparin	2b	3	N.A.	N.A.	−
	Case series	L ICA	L M1	3.9	Stent	Merci stent retriever + heparin + IA thrombolytics	2a	1	14	N.A.	+
	Case series	L ICA	L M2	2.8	None	Merci stent retriever + heparin	2b	3	28	N.A.	−
	Case series	R ICA	R intracranial ICA, R M1	3.1	None	Merci stent retriever	3	0	19	N.A.	+
	Case series	R ICA	R M1/M2	7.4	Stent^†^	Merci stent retriever + IA thrombolytics	0	2	11	N.A.	−
	Case series	L ICA	R M1/M2	3.2	None	Merci stent retriever + IA thrombolytics	3	0	14	N.A.	+
	Case series	L ICA	L ICA/M2	6.3	None	Merci stent retriever + IA thrombolytics	0	1	1	N.A.	+
	Case series	L ICA	L M1/M2	2.7	None	Merci stent retriever + IA thrombolytics	2b	2	23	N.A.	+
Fujimoto et al. [26]	Case report	R intracranial ICA	R M1/M2/A1	5.2	None	032 Penumbra System	2a	N.A.	20	1	+
Kondziella et al. [29]	Review article	R ICA	R M1	4.0	Carotid Wallstent	Solitaire stent retriever	2b	1	22	1	+
Kulcsár et al. [17]	Case report	L ICA	L M1	4.2	Enterprise stent^‡^	Heparin + IV aspirin	2a	4	16	N.A.	−
Lavallée et al. [1] (6 cases)	Case-control	ICA	M1	5.6	Carotid Wallstent	IA thrombolytics + clopidogrel + aspirin + heparin	1	2	19	6	+
	Case-control	ICA	M1	4.6	Carotid Wallstent	IA thrombolytics + clopidogrel + aspirin + heparin	2a	0	14	0	+
	Case-control	ICA	M1	3.4	Carotid Wallstent	IA thrombolytics + clopidogrel + aspirin + heparin	2a	0	16	0	+
	Case-control	ICA	M1	3.8	Carotid Wallstent	IA thrombolytics + clopdiogrel + aspirin + heparin	2a	0	12	0	+
	Case-control	ICA	M1	6.0	Carotid Wallstent	IA thrombolytics + clopdiogrel + aspirin + heparin + mechanical thrombectomy	2a	0	17	0	+
	*n*	ICA	M1	5.9	Carotid Wallstent	IA thrombolytics + clopdiogrel + aspirin + heparin	2a	2	20	4	+
Lekoubou et al. [18] (3 cases)	Case report	R extracranial ICA	R M1	4.3	Leo stent	IA thrombolytics + clopidogrel + aspirin + heparin	N.A.	2	14	N.A.	+
	Case report	R extracranial ICA	R M1	4.0	Leo stent	IA thrombolytics + clopdiogrel + aspirin + heparin	N.A.	0	6	N.A.	+
	Case report	R extracranial ICA	L PCA	2.2	Leo stent	IA thrombolytics + clopdiogrel + aspirin + heparin	N.A.	1	16	N.A.	+
Lescher et al. [19]	Case series	extracranial ICA (7 points)	N.A.	5.6	Carotid Wallstent	Clopidogrel + aspirin	4/7 ≥ 2b	0–2 in 4/7	15	N.A.	+ 4/7
Lockau et al. [20]	Review article	ICA (13 points)	ACA or MCA/distal ICA	1.8	Carotid Wallstent	IA thrombolytics + clopdiogrel + aspirin	10/13 2b/3	0–2 in 8/13	18	N.A.	N.A.
Mourand et al. [21] (2 cases)	Case report	R extracranial ICA	R intracranial ICA, R MCA	4.0	Enterprise stent (2×)	IA thrombolytics + heparin	2a	0	15	0	+
	Case report	R extracranial ICA	R M1/M2, R A1	4.0	Wingspan stent	IA thrombolytics + heparin	3	1	18	0	+
Padalino et al. [25] (2 cases)	Case report	L extracranial ICA	L MCA	6.5	Acculink stent + balloon angioplasty	IA abciximab + mechanical thrombectomy +026 Penumbra system	3	N.A.	13	11	−
	Case report	L extracranial ICA	L MCA	6.1	Acculink stent	IA abciximab + mechanical aspiration +026 Penumbra system	3	N.A.	22	0	+
Jensen et al. [23]	Case-control	Extracranial ICA (17 points)	N.A. but yes	N.A.	Stent + angioplasty	IA thrombectomy + IA thrombolytics	16/17 2b/3	0–2 in 13/17	13	N.A.	+ 13/17
Cohen et al. [22] (6 cases)	Case series	L extracranial ICA	L ICA ‘T’	1.0	Carotid Wallstent	IV heparin + Solitaire stent retriever + clopidogrel	2b	0	24	N.A.	+
	Case series	L extracranial ICA	M1	1.2	Carotid Wallstent	IV heparin + Solitaire stent retriever + clopidogrel	3	1	22	N.A.	+
	Case series	L extracranial ICA	L ICA T	−	Carotid Wallstent	IV heparin + Solitaire stent retriever + clopidogrel	1	3	24	N.A.	−
	Case series	L extracranial ICA	M1	0.8	Carotid Wallstent	IV heparin + Solitaire stent retriever + clopidogrel	3	1	12	N.A.	+
	Case series	R extracranial ICA	M1	0.8	Carotid Wallstent	IV heparin + Solitaire stent retriever + clopidogrel	3	1	12	N.A.	+
	Case series	L extracranial ICA	M1-M2	1.0	Carotid Wallstent	IV heparin + Solitaire stent retriever + clopidogrel	2b	2	20	N.A.	+
Marnat et al. [24]	Case-control	extracranial ICA (20 points)	M1, M1-M2	4.5	Carotid Wallstent + Leo stent	IV aspirin + clopidogrel	14/20 2b/3	0–2 in 14/20	18	N.A.	+14/20

*L* left, *R* right, *TTT* time to treatment, *BL* baseline

^†^extension of a dissected carotid artery by a microcatheter – was treated effectively with stenting; ^‡^craniectomy

### Complications during procedures

In one case [17], decompressive craniectomy was performed after endovascular therapy and one case described an extension of the dissection caused by a microcatheter [2]. This extension was treated effectively with stenting.

### Reperfusion outcome data

Not all included studies described the reperfusion outcomes. Reperfusion outcome data were described in 16 studies. TICI 2b-3 was described in 10 studies. In only a few studies, TICI 2a was reported after recanalisation. Even less studies presented patients with very limited reperfusion. For individual TICI data, see Table 1.

### Clinical outcome data

As displayed in Table 1, most studies showed favourable clinical outcome and successful reperfusion. Eleven out of 12 studies that described clinical outcome using 90-day mRS showed favourable clinical outcome. Seven out of eight studies that described 90-day NIHSS as an outcome parameter showed a favourable clinical outcome.

## Discussion

Most included studies on endovascular treatment in patients with proximal intracranial occlusion and a concomitant ICA dissection present favourable clinical outcome and successful reperfusion. A favourable 90-day NIHSS was found in the majority of cases in which this parameter was denoted. Besides, favourable 90-day mRS was found for most of the cases for patients of which the follow-up was classified by mRS. The vast majority of treated patients had successful reperfusion. However, it should be noted that the risk of bias in these studies is high.

A similar study [30] indicated that endovascular treatment had more beneficial outcomes than intravenous treatment in patients with ICA dissection-related stroke. Yet, in this study, there was no differentiation between patients with an exclusive ICA dissection and patients with both an ICA dissection and concomitant occluded intracranial artery. The fact that this study also included patients with an ICA dissection without an additional intracranial occlusion might explain why they found a higher percentage of patients with favourable reperfusion. The percentage of cases with favourable clinical outcome was comparable to our series.

Another recent study on RECOST study data [24] showed that a distal to proximal treatment strategy of ICA dissection with a concomitant intracranial occlusion appears safe and effective [24]. It is argued in this study that a conservative approach (considering stent placement only after intracranial revascularisation in case of circle of Willis insufficiency) may be reliable and safe. However, the RECOST study included only 20 patients with an ICA dissection together with an intracranial occlusion. Hence, beneficial outcome of a conservative approach has only been researched in a small number of patients.

Regarding the risk of bias of included studies, most of the included studies were retrospective case reports or case series (see Table 1). By definition, this led to a high sensitivity to bias. It is possible that only cases with favourable clinical outcome rates and successful reperfusion were published. This could also explain why outcomes found in our review are substantially more positive than the outcomes in the held RCTs [6–12].

Data in the included studies was not always consistently described. Some studies only used mean values of all included patients and patient characteristics were not always clearly described. This could have influenced the accuracy of our results, since individual results are left out.

Only a small number of patients received treatment of the intracranial occlusion with second-generation thrombectomy devices (Solitaire or Penumbra thromboaspiration system). All other included cases described the use of currently obsolete techniques. Unfortunately, studies that describe results of second-generation treatment methods are not widely available yet.

Furthermore, time to treatment in the reviewed studies was relatively long. This could be due to the fact that the procedural time that is needed to stent the ICA increases the onset-to-reperfusion time. This applies for a treatment strategy with first performing recanalisation in the ICA and secondly in the intracranial occlusion. The relatively long TTT could possibly be explained by the fact that most patients were initially not presented to the hospital with a dissection. The additional finding of ICA dissection in the emergent setting and the subsequent need for an experienced endovascular therapist might have delayed the procedure. No statistics on the collected data could be performed since the patient characteristics and data were non-poolable. This is because included patients did not have similar baseline characteristics, different types of treatment were used, and data measurement varied between studies.

The high success rates reported in the included studies suggest that endovascular treatment is safe and efficacious for patients with a proximal occlusion with a concomitant ICA dissection. However, we need to stress that the number of patients included in this review is low and the risk of bias is high. Therefore, further research is needed.

## Conclusion

High rates of favourable outcome and reperfusion have been presented in reviewed studies. This suggests that endovascular treatment is beneficial in patients with ICA dissection and concomitant proximal intracranial occlusion. This corresponds with earlier emerged studies that showed that mechanical thrombectomy is beneficial in patients with large vessel occlusion. However, this review has only taken data of a limited group of patients into account. A pooled analysis of patients with ICA dissection and concomitant proximal intracranial occlusion from the recently published endovascular treatment trials and running registries is therefore recommended.

## Electronic supplementary material

Table S1 (PDF 512 kb)

Figure S1 (PDF 408 kb)

Figure S2 (PDF 394 kb)
