# Supplementary material for: Endovascular treatment in patients with carotid artery dissection and intracranial occlusion: a systematic review
Source: Neuroradiology. 2017 Jun 3;59(7):641–7. doi: 10.1007/s00234-017-1850-y (PMC5493704; doi:10.1007/s00234-017-1850-y)
Supplement: Supplementary file 1 — (PDF 512 kb) [file 234_2017_1850_MOESM1_ESM.pdf]

Table 4 *Cochrane Risk of Bias Tool – Analysis*

STUDY	RANDOM SEQUENCE GENERATION	ALLOCATION CONCEALMENT	BLINDING PATIENTS AND PERSONNEL	BLINDING OUTCOME ASSESSMENT	INCOMPLETE OUTCOME DATA	SELECTIVE REPORTING	OTHER BIAS
**Baumgartner et al**	High	Unclear	High	Low	Low	Low	High
**Bulsara et al**	High	Unclear	High	Low	Low	Low	Unclear
**Cohen et al**	High	Unclear	High	Low	Low	Low	High
**Sainz de la Maza et al**	High	Unclear	High	Low	High	Low	High
**Fields et al**	High	Unclear	High	Low	Low	High	High
**Fujimoto et al**	High	Unclear	High	Low	Low	Low	High
**Jensen et al**	High	Unclear	High	Low	High	High	High
**Kondziella et al**	Low	Unclear	Low	Unclear	Low	Unclear	Low
**Kulcsár et al**	High	Unclear	High	Low	Low	High	Unclear
**Lavallée et al**	High	Unclear	High	Low	Low	Low	High
**Lekoubou et al**	High	Unclear	High	Low	High	High	High
**Lescher et al**	High	Unclear	High	Low	High	High	High
**Lockau et al**	High	Unclear	High	Low	High	High	High
**Marnat et al**	High	Unclear	High	Low	High	High	High
**Mourand et al**	High	Unclear	High	Low	High	High	High
**Padalino et al**	High	Unclear	High	Low	High	High	High
