# Supplementary figures and images for: Endovascular treatment in patients with carotid artery dissection and intracranial occlusion: a systematic review
Source: Neuroradiology. 2017 Jun 3;59(7):641–7. doi: 10.1007/s00234-017-1850-y (PMC5493704; doi:10.1007/s00234-017-1850-y)

**Fig.1** *Cochrane Risk of Bias Tool – Graph*

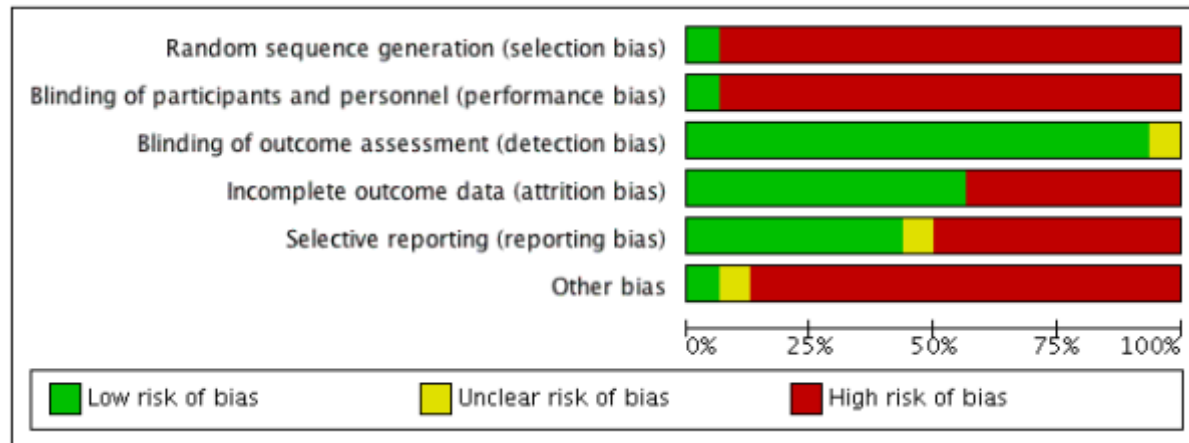

Supplement: Supplementary file 2 — (PDF 408 kb) [file 234_2017_1850_MOESM2_ESM.pdf]
