# Supplementary material for: Endovascular treatment in patients with carotid artery dissection and intracranial occlusion: a systematic review
Source: Neuroradiology. 2017 Jun 3;59(7):641–7. doi: 10.1007/s00234-017-1850-y (PMC5493704; doi:10.1007/s00234-017-1850-y)
Supplement: Supplementary file 3 — (PDF 394 kb) [file 234_2017_1850_MOESM3_ESM.pdf]

Fig.2 *Cochrane Risk of Bias Tool – Table*

	Random sequence generation (selection bias)	Blinding of participants and personnel (performance bias)	Blinding of outcome assessment (detection bias)	Incomplete outcome data (attrition bias)	Selective reporting (reporting bias)	Other bias
Baumgartner 2008	−	−	+	+	+	−
Bulsara 2013	−	−	+	+	+	−
Cohen 2015	−	−	+	+	+	−
Fields 2012	−	−	+	+	−	−
Fujimoto 2013	−	−	+	+	+	−
Jensen 2016	−	−	+	−	−	−
Kondziella 2013	+	+	?	+	?	+
Kulcsar 2010	−	−	+	+	−	?
Lavallee 2007	−	−	+	+	+	−
Lekoubou 2010	−	−	+	−	−	−
Lescher 2015	−	−	+	−	−	−
Lockau 2015	−	−	+	−	−	−
Marnat 2016	−	−	+	−	−	−
Mourand 2010	−	−	+	+	+	−
Padalino 2012	−	−	+	−	−	−
Sainz de la Maza 2014	−	−	+	−	+	−
